# Case Report: Kikuchi-Fujimoto disease: a diagnostic and therapeutic dilemma following pretransplant nephrectomy for a 2.35 Kg kidney

**DOI:** 10.12688/f1000research.8992.1

**Published:** 2016-06-17

**Authors:** Arvind P. Ganpule, Jaspreet Singh Chabra, Abhishek G. Singh, Gopal R. Tak, Shailesh Soni, Ravindra Sabnis, Mahesh Desai

**Affiliations:** 1 Muljibhai Patel Urological Hospital, Nadiad, Gujarat, 387001, India

**Keywords:** Kikuchi Fujimoto disease, Autosomal dominant polycystic kidney disease, Lymphadenopathy

## Abstract

Kikuchi-Fujimoto disease (KFD) is an extremely rare disease with a worldwide distribution and higher prevalence in Asians. It is a benign and self-limiting disorder, characterized by regional cervical lymphadenopathy accompanied with mild fever and night sweats. Lymph node histopathology is diagnostic and treating physicians should be aware of this entity as it may mimic other systemic diseases like systemic lupus erythematosus, tuberculosis, malignant lymphoma, and more rarely adenocarcinoma. Key features on lymph node biopsy are fragmentation, necrosis and karyorrhexis.

Treatment includes symptomatic care, analgesics-antipyretics, corticosteroids and spontaneous recovery occurs in 1 to 4 months. We report a case of adult polycystic kidney disease (ADPKD) with end stage renal disease and episodes of fever and cervical lymphadenopathy. The infectious screen was negative and on extensive workup, the patient was found to have histiocytic-necrotizing lymphadenitis, which clinched the diagnosis of KFD.

## Introduction

Kikuchi-Fujimoto disease (KFD), or histiocytic necrotizing lymphadenitis, is a benign and self-limiting disease that mainly affects young women. Recognition of this condition is critical as it can mimic tuberculosis, lymphoma, or even adenocarcinoma. Awareness of this entity helps to prevent misdiagnosis and inappropriate treatment ^1^.

The aim of this report is to review the authors' institutional experience of KFD in a patient with end stage renal disease being prospectively evaluated for renal allograft transplant. Diagnosis of KFD should be kept in mind in patients who present with fever and cervical lymphadenopathy. This presentation may not be a rarity in immunocompromised pre and post transplant patients. We report a case of KFD in a patient with adult polycystic kidney disease who underwent bilateral pre-transplant nephrectomy.

Keywords: Kikuchi – Fujimoto disease (KFD), Autosomal dominant polycystic kidney disease (ADPKD), Lymphadenopathy

## Case history

A 42 years old Asian male on evaluation of abdominal pain was diagnosed with adult polycystic kidney disease (ADPKD) in 2002. The patient’s mother died of renal failure due to ADPKD. Patient had progressive deterioration of renal function and developed chronic kidney disease (CKD) stage 5 in 2015. The patient was advised renal replacement therapy (RRT) and required thrice-weekly hemodialysis since September 2015. He was evaluated for renal transplant and planned for the same in May 2016. During pre-transplant evaluation he developed fever with chills and bilateral painful cervical lymphadenopathy in the posterior triangle of neck. Ultrasound of cervical region reported presence of multiple enlarged lymph nodes in the neck along internal jugular vein on both the sides; largest node on right side measured 21×7 mm in size and on the left side largest node measured 16×5 mm in size. Ultrasound examination of axilla and groin did not reveal lymphadenopathy.

The patient underwent right cervical lymph node biopsy. Histopathology was reported to be acute necrotizing lymphadenitis. This biopsy was negative for acid-fast bacilli (AFB) and fungal stains. In the meanwhile his complete infectious screen of blood, urine, sputum and stool cultures showed no growth. The patient was given broad-spectrum third generation cephalosporins for 14 days assuming the fever to be caused by unidentifiable bacterial infection.

**Figure 1.**
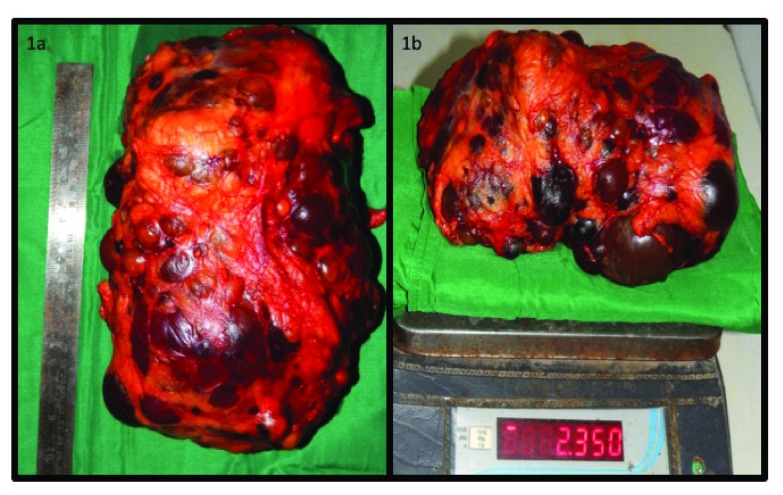
**Figure 1a** is showing length of the specimen in cm and **Figure 1b** shows weight of the specimen in Kg on a certified weighing scale.

The fever settled and the patient was planned for a bilateral pre-transplant nephrectomy in view of bilateral large size kidney and recurrent fever. Right laparoscopic Pre-transplant nephrectomy was done on 05 February 2016. This was followed by a pre-transplant left open nephrectomy on 16 February 2016. The left side was planned for an open nephrectomy in view of the large size of the kidney. The procedure was uneventful. Incidentally, this was one of the largest kidney specimen ever recorded in literature weighing 2.35 Kg ( Figure 1a & 1b). The Guinness Book of World Records reports a kidney weighing 2.15 kg as world's largest kidney till now which was retrieved in Dhule, Maharashtra, India ^2^.

Postoperatively, the patient had continuous fever of 100 degree Fahrenheit with chills for 10–15 days; despite he receiving third generation broad-spectrum injectable antibiotics. As the patient had undergone a bilateral nephrectomy, the first diagnosis for pyrexia of unknown origin was postoperative surgical infection or intra-abdominal abscess formation. The imaging done included high-resolution computerized tomography (CT) chest (HRCT chest), CT abdomen and CT pelvis. These did not reveal any significant finding. His fever did not subside despite all the measures such as higher antibiotics, antipyretics and removal of all indwelling catheters. His indwelling catheter lines were cultured to rule out any infection, but did not grow any organism. An infectious disease specialist was consulted and he advised for a slide review of the cervical lymph node sample, which was done prior to nephrectomy.

**Figure 2.**
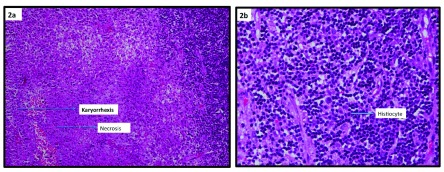
**Figure 2a** showing low power light microscopic view of lymphnode biopsy highlighting karyorrhexis and necrosis. **Figure 2b** showing a high power light microscopy images of cervical lymphnode biopsy demonstrating histiocytes.

Slide review with the specialist suggested a necrotizing histiocytic lymphadenitis (Kikuchi Disease) ( Figure 2a & 2b). Section revealed lymphoid architecture being replaced by necrotizing lesions, which are composed of karyorrhectic debris with fibrin deposits and collection of mononuclear cells. Plasma cells and neutrophils were scanty. Periodic acid-Schiff (PAS) stain was negative for fungal hyphae. Ziehl Neelsen (ZN) stain was negative for AFB with final diagnosis as necrotizing histiocytic lymphadenitis. Tests for bacteria/fungus/AFB were negative. Genexpert examination for tubercle bacilli (TB) was also negative. Immmunohistochemistry (IHC) examination for lymphoma was negative. Tests for systemic lupus erythematosus (SLE) were also negative. The key features which clinched the histological diagnosis were: karyorrhexis, fragmentation and necrosis.

The patient was started on non-steroidal anti-inflammatory drugs (NSAID), oral Prednisolone 10 mg twice a day for 15 days and continued on hemodialysis (HD). Patient responded well to this treatment measures. A team of an infection specialist, a nephrologist and a urologist managed this patient. The patient finally became afebrile and fit for renal transplantation after a treatment for 3 weeks. He underwent a living related renal transplant on 27 ^th^ of April 2016. His postoperative course after renal transplantation was uneventful and achieved a nadir creatinine of 1.05 mg/dl.

## Discussion

KFD, or histiocytic necrotizing lymphadenitis, was originally reported in 1972 in Japan ^3^. KFD is a rare disease, more commonly occurring in females of Asian origin during the third decade of life, although it has been reported between 19 months to 75 years of age. Association of KFD with HLA class II alleles (HLA-DPA1 and HLA-DPB1 especially) has been described in Asian KFD patients ^4^.

Incidence of KFD is higher in females with the most common presentation being cervical lymphadenopathy and with half of patients presenting fever with leucopenia ^5– 7^. Patients suspected to have this disease should be extensively examined and tested for tuberculosis, Epstein-Barr virus, cytomegalovirus, human immunodeficiency virus and SLE. Lymphoma can also be mimicked by this disease and has to be ruled out by immmunohistochemistry. Key histological features of KFD include single or focal areas within lymph node containing histiocytic cellular infiltrate with necrosis, with perinodal inflammation and occasional capsular invasion ^8^.

The signs and symptoms of Kikuchi disease are fever, lymphadenopathy, skin rashes and headache. Rarely, hepato-splenomegaly and nervous system involvement resembling meningitis are seen. This disease is also associated with bouts of extreme fatigue, especially during latter parts of the day.

Differential diagnosis of KFD includes SLE, disseminated tuberculosis, lymphoma, sarcoidosis and viral lymphadenitis. Clinical findings sometimes may include positive results for IgM/IgG/IgA antibodies for respective diseases. In contrast to published reports of KFD, which showed a female predominance, in our case it occurred in a male patient.

## Conclusion

KFD, although rare, should be part of the differential diagnosis in patients presenting with fever and cervical lymphadenopathy in chronic kidney disease patients, especially in patients of Asian origin who presents a negative infectious screen.

Key messages from our case report:

1) High degree of suspicion is required for diagnosis of KFD. KFD is a self-limiting benign disorder characterized by lymphadenopathy, which resolves in a few weeks to months.
2) The pillars for diagnosis for KFD include histological features of necrosis, fragmentation and karyorrhexis in lymph node biopsy.
3) In immunosuppressed patients as in this case, the diagnostic dilemma remains and the differential diagnosis to be considered include systemic lupus erythematosus (SLE), disseminated tuberculosis, lymphoma, sarcoidosis, and viral lymphadenitis.
4) Apart from the work up of pyrexia of unknown origin, an experienced histopathologist should do a slide review of cervical lymphnode biopsy.
5) The management should be multimodal; as in this case it involved a nephrologist, an urologist and an infection disease specialist. Treatment is mostly supportive and steroids may be helpful in the treatment.

## Patient consent

Written informed consent for publication of clinical details and clinical images was obtained from the patient.
