# Supplementary material for: Case Report: Kikuchi-Fujimoto disease: a diagnostic and therapeutic dilemma following pretransplant nephrectomy for a 2.35 Kg kidney
Source: F1000Res. 2016 Jun 17;5:1407. [Version 1] doi: 10.12688/f1000research.8992.1 (PMC4926730; doi:10.12688/f1000research.8992.1)
Supplement: Peer review history

# Peer review: Referee response for version 1

**DOI:** 10.5256/f1000research.9673.r14562

**Published:** 2016-06-24

**Authors:** Sanjay Kulkarni

**Affiliations:** 1 Kulkarni Reconstructive Urology Center, Pune, India

This is an interesting case report. KFD is a treatable but rare disorder. In this particular patient it was diagnosed due to a vigilant clinician and pathologist. KFD should be kept in differential diagnosis.

 I recommend that this article is indexed.

I have read this submission. I believe that I have an appropriate level of expertise to confirm that it is of an acceptable scientific standard.

# Peer review: Referee response for version 1

**DOI:** 10.5256/f1000research.9673.r14541

**Published:** 2016-06-23

**Authors:** Noor NP Buchholz

**Affiliations:** 1 Department of Urology, Sobeh’s Vascular & Medical Center, Dubai, 505008, United Arab Emirates

The article is well written and indeed presents an extremely rare occurrence. I have little to add, I would recommend indexing.

 I would amend the bit about the Guinness World Record, Medicine is not a contest.

I have read this submission. I believe that I have an appropriate level of expertise to confirm that it is of an acceptable scientific standard.
